# Human Serum Albumin (HSA) Suppresses the Effects of Glycerol Monolaurate (GML) on Human T Cell Activation and Function

**DOI:** 10.1371/journal.pone.0165083

**Published:** 2016-10-20

**Authors:** Michael S. Zhang, Jon C. D. Houtman

**Affiliations:** Department of Microbiology, Carver College of Medicine, University of Iowa, Iowa City, Iowa 52242, United States of America; Islamic Azad University Mashhad Branch, ISLAMIC REPUBLIC OF IRAN

## Abstract

Glycerol monolaurate (GML) is a monoglyceride with well characterized anti-microbial properties. Because of these properties, GML is widely used in food, cosmetics, and personal care products and currently being tested as a therapeutic for menstrual associated toxic shock syndrome, superficial wound infections, and HIV transmission. Recently, we have described that GML potently suppresses select T cell receptor (TCR)-induced signaling events, leading to reduced human T cell effector functions. However, how soluble host factors present in the blood and at sites of infection affect GML-mediated human T cell suppression is unknown. In this study, we have characterized how human serum albumin (HSA) affects GML-induced inhibition of human T cells. We found that HSA and other serum albumins bind to 12 carbon acyl side chain of GML at low micromolar affinities and restores the TCR-induced formation of LAT, PLC-γ1, and AKT microclusters at the plasma membrane. Additionally, HSA reverses GML mediated inhibition of AKT phosphorylation and partially restores cytokine production in GML treated cells. Our data reveal that HSA, one of the most abundant proteins in the human serum and at sites of infections, potently reverses the suppression of human T cells by GML. This suggests that GML-driven human T cell suppression depends upon the local tissue environment, with albumin concentration being a major determinant of GML function.

## Introduction

Glycerol monolaurate (GML) is composed of a glycerol head group with one fully saturated 12-carbon medium chain fatty acid. GML potently suppresses the growth of a wide spectrum of pathogens, including gram positive and negative bacteria, fungi, and enveloped viruses [1–6]. Due to these antimicrobial properties, GML is incorporated in numerous commercial products such as deodorants, lotions, cosmetics, foods, and homeopathic supplements [7–9]. GML is also currently being tested as a topical therapeutic for toxic shock syndrome, HIV transmission, and surgical site infections [10–12]. Thus, the commercial and clinical use of GML is greatly expanding. Interestingly, the antimicrobial properties of GML may act at sites distal to the administered site. Rodents orally fed with GML have reduced *Staphylococcus Aureus* induced peritonitis disease burden in the abdominal cavity at levels comparable to rodents fed with vancomycin, suggesting that GML potentially binds to and be carried by soluble factors to distal sites [13,14].

In addition to its anti-microbial actions, several studies have shown that GML also modulates the immune system. GML suppresses mitogen- and superantigen-driven lymphocyte proliferation and IP_3_ levels, a key messenger molecule in T cell signal transduction [15,16]. We have recently described that GML treated human T cells have altered dynamics of ordered lipid domains in the plasma membrane. This dysregulation of membrane homeostasis inhibits the aggregation of LAT, PLC-γ1, and AKT nanoclusters into microcluster units at the plasma membrane. The lack of microcluster formation of these signaling molecules results in suppressed TCR activation-induced calcium influx, PI3K and AKT phosphorylation, and cytokine production [17]. Thus, GML is a powerful pharmacologic agent with both antimicrobial and immunosuppression properties.

Despite the extensive use of GML in numerous commercial and clinical products, how GML interacts with soluble factors in humans has not been investigated. GML will encounter a wide array of molecules at distinct concentrations depending on its application in the gut, skin, or vaginal tract. One protein that is present in substantial concentrations across all the aforementioned tissue sites is human serum albumin (HSA). HSA is the most abundant protein in the circulatory plasma with a wide array of functions. At a serum concentration of 35–50 g/L, HSA is the main protein component responsible for sustaining colloidal osmotic blood pressure [18]. Additionally in superficial skin inflammation, the concentration of HSA in the skin increases to levels comparable to serum concentrations at 10–30 g/L [19]. HSA is capable of binding to various endogenous organic compounds such as fatty acids, steroids, tryptophan, bilirubin, etc as well as inorganic molecules including copper, zinc, calcium, and others [18,20]. HSA is important in pharmacokinetics due to its ability to bind and modulate the pharmacologic activity of exogenous drugs including warfarin, ibuprofen, chlorpromazine, and naproxen [18,20,21]. The interaction between HSA and its ligands has profound impact on the chemistry of the ligands. For example, fatty acids and copper bound to HSA have reduced redox chemistry and free radical formation [18,20]. Additionally, displacement of warfarin from HSA by other HSA ligands is known to significantly increase the drug’s bioavailability and toxic side effects [22]. Hence, HSA is a critical host factor in modulating the chemical activity of a wide variety compounds, including fatty acids. Due to the fatty acid chemical structure of GML, we hypothesized GML interacts with HSA and alters GML induced suppression of human T cells.

To address the question of how HSA affects GML-induced T cell suppression, we characterized the binding affinity between HSA and GML and investigated how HSA alters GML mediated suppression of human T cell signaling events and effector cytokine production. We found that HSA has a relatively strong binding affinity for GML at low micromolar dissociation constants (Kds). This interaction between GML and HSA mitigated the signaling defects in GML treated cells. Namely HSA allows the formation of AKT, LAT, and PLC-γ1 microclusters, restores AKT phosphorylation at threonine 308 and serine 473, and rescues IFN-γ, IL-2, IL-10, and TNFα production in GML-treated cells. These results show that the degree of T cell suppression by GML is inversely correlated with the HSA concentration. This information provides insight into physiologic scenarios that GML is expected to be T cell suppressive, information that is critical for the proper utilization of GML in its various applications.

## Materials and Methods

### Primary human T cell isolation and GML preparation

All human subjects experiments were performed in accordance with the guidelines set forth in the Declaration of Helsinki. Peripheral blood mononuclear cells (PBMCs) were isolated from the whole blood of healthy donors that have consented for blood donation at the DeGowin Blood Center at the University of Iowa Hospitals and Clinics. Donors are anonymous and have provided written informed consent to allow their cells that are normally discarded to be used in research studies. The recruitment protocol and written informed consent document were approved by the Institutional Review Board for the University of Iowa. All samples were provided to investigators de-identified. Therefore, further IRB approval for the use of the cells by the investigators was not needed based on Federal Regulation 46.101.B4. PBMCs were isolated using Hypaque-Ficoll density-gradient separation. T cells were then expanded and activated using anti-CD3 and anti-CD28 coated beads (Invitrogen) and human IL-2 for five days. Cells were then re-suspended in fresh media without stimulatory beads and IL-2 for 24 hours and are termed activated peripheral blood T cells (APBTs). APBTs were resuspended in serum free media or media supplemented with fatty acid free HSA (Sigma) before GML treatment. GML was solubilized at room temperature in 95% ethanol and diluted into the appropriate working concentration. 95% ethanol was added as comparative vehicle control at final concentrations that did not exceed 0.2%.

### Enzyme-linked immuno assay (ELISA) detection of cytokines

0.2% Ethanol control or various doses of GML were added to APBTs in serum free media or media supplemented with between 0.2% to 2% HSA. The cells were then stimulated with plate-bound 2 μg/ml of anti-CD3 (Biolegend) for 24 hours. Protein concentrations of IL-2, IFN-γ, IL-10, and TNF-α in the media supernatants were measured by standard TMB ELISA.

### Immunoblotting

APBTs were treated with 0.1% ethanol control or 10 μg/ml GML resuspended in serum free media or media supplemented with 1% HSA. The cells were then incubated on ice with 2 μg/ml anti-CD3 for 30 minutes. Cells were then warmed at 37°C for 10 minutes, stimulated using IgG crosslinking antibody for various times and HSA was washed away via centrifugation. Cells were then lysed with 2X sample buffer, heated to 95°C, and sonicated.

Cell lysates were separated by polyacrylamide gel electrophoresis and transferred to PVDF. The membranes were blocked in 0.5X SEA BLOCK blocking buffer diluted in PBS. Primary antibodies were incubated overnight at 4°C. Secondary antibodies were incubated with the membrane for 30 minutes at room temperature and then imaged using Licor Odyssey (Lincoln, NE, USA). At least four independent replicates with different human donors were performed for each experiment. Immunoblot band intensity was quantified using Odyssey’s v3.0 software with the intensity of the phospho-specific antibody normalized to the intensity of the GAPDH antibody. To minimize variability between human donors, band intensities were further normalized to maximal band intensity of ethanol vehicle control. The following antibodies were used for immunoblotting: AKT pThr 308 (Cell Signaling), AKT pS473 (Invitrogen), GAPDH (Meridian Life Sciences), and IRDye 800CW or IRDye680-conjugated secondary antibodies (Thermo Scientific).

### Total Internal reflection fluorescence (TIRF) microscopy

APBTs were stimulated in the presence of 0.1% ethanol control or 10 μg/ml GML with plate-bound anti-CD3 on glass coverslips, fixed with 4% paraformaldehyde, permeabilized with 0.25% Triton-X, and stained as described below. To detect microcluster formation of TCR signaling proteins, antibodies specific for LAT pY226 (BD Pharmingen), PLC-γ pY783 (Cell Signaling, and total AKT (Cell signaling) were incubated with fixed and permeabilized cells overnight for 4°C. Subsequently, conjugated secondary antibodies Dylight Goat anti-rabbit IgG and DyLight 568 Goat anti-rabbit IgG (Biolegend) as well as Alexa Fluor 488 goat anti-mouse IgG1 (Thermo Fisher) were incubated with the cells at room temperature for 2 hours. All images were captured by the Leica AM TIRF MC system using 100x magnification oil immersion objective lens at room temperature at the University of Iowa Central Microscopy Research Facility.

### Image quantification

TIRF microscopy images were processed and analyzed using ImageJ. Quantification of membrane clustering was done by measuring mean pixel intensity in the longest axis of cells for 60 cells total from at least 3 independent experiments. Similar quantification methods have been used in other published reports [23–25].

### Isothermal Titration Calorimetry and Ligand Binding Analysis

HSA, bovine serum albumin (BSA), pig serum albumin (PSA), rabbit serum albumin (RSA), and mouse serum albumin (MSA) were purchased from Sigma Aldrich and purified using gel filtration in 10 mM sodium phosphate and 150 mM potassium chloride buffer at pH 7.4 at room temperature. Lauric acid (LA) was purchased from Cayman Chemicals and glycerol was purchased from RPI. Final protein concentrations were adjusted to 5 μM for all albumin proteins, and final GML, LA, and glycerol concentration was 150 μM. All samples were degassed, and ITC was performed at room temperature as previously described using MicroCal VP-ITC calorimeter [26]. Albumin proteins were placed in the sample cell and GML, LA, and glycerol were in the syringe. Twenty-one injections of GML, LA, or glycerol were done at intervals of 180 seconds. Control experiments where GML, LA, or glycerol was injected into buffer and ethanol control was injected into albumin proteins showed that the heats of dilution were constant across all injections. The data were analyzed using a single-site binding model provided in the ITC analysis software package. To ensure reproducibility, three independent experiments from three different preparations of HSA and GML were carried out. The values for ΔH, K_d_ and stoichiometry averaged ± standard deviation. The percent of GML bound to HSA or the percent of HSA bound to GML was calculated using the standard mass action equation with the assumptions that HSA will bind to three GML molecules, that all three binding sites on HSA had an equivalent affinity for GML and that no other ligands are present with GML. The values for stoichiometry and binding affinity were derived from the ITC experiments.

### Statistical Analysis

Student’s t test was done in Microsoft excel by use of two-tailed test assuming equal variance.

## Results

### HSA binds to the saturated 12 carbon acyl chain component of GML with high affinity and this interaction affects the concentration of HSA-GML complexes in solution

**Fig 1:**
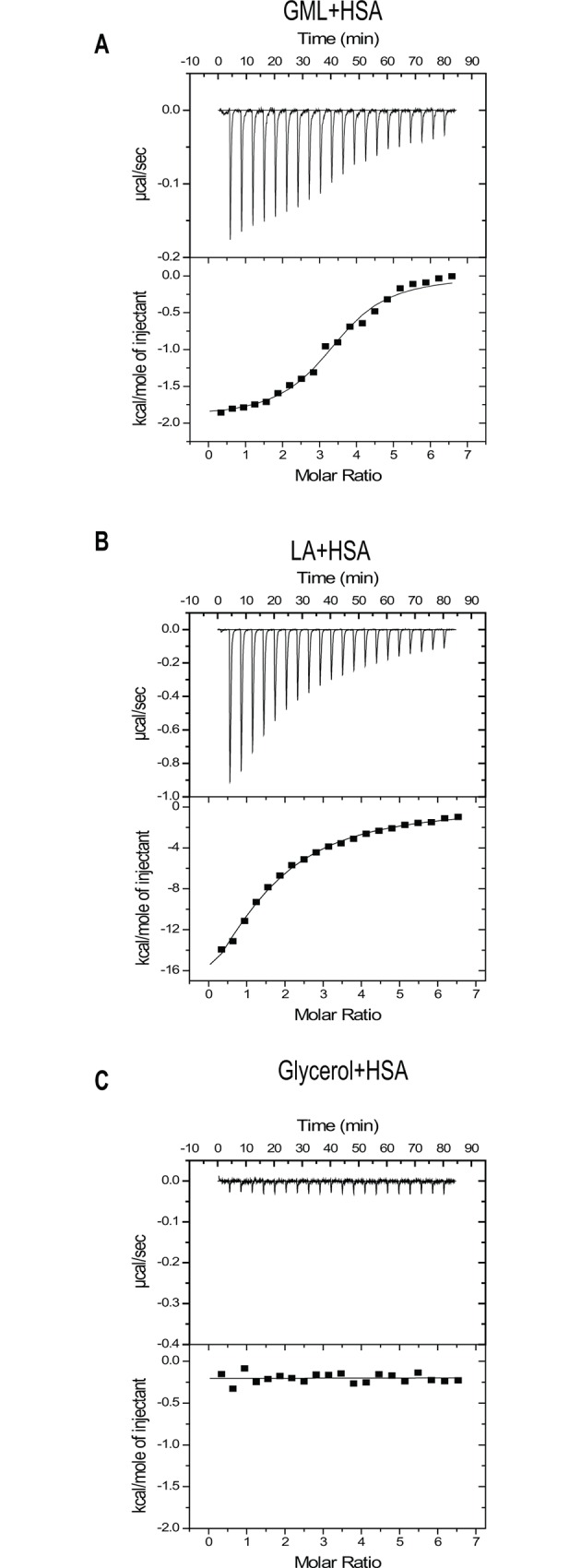
HSA interacts with GML via high affinity binding to the saturated 12 carbon chain. **(A)** 5μM of HSA were purified via gel filtration and placed in the sample chamber of the ITC. 150 μM of ethanol solubilized GML was placed in the syringe and injected into the sample chamber twenty one times with 180 seconds interval between injections. Top graph shows the change of power with each GML injection and bottom graph shows data fitted with a single binding site model for GML and HSA. **(B)** 5μM of gel filtrated HSA was placed in the sample chamber of the ITC and 150 μM of ethanol solubilized lauric acid (LA) was placed in the syringe that was injected into the sample chamber twenty one times with 180 seconds interval between injections. Top graph shows the change of power with each LA injection and bottom graph shows data fitted with a single binding site model for GML and LA. **(C)** 5μM of gel filtrated HSA was placed in the sample chamber of the ITC and 150 μM of glycerol was placed in the syringe that was injected into the sample chamber twenty one times with 180 seconds interval between injections. Top graph shows the change of power with each glycerol injection and bottom graph shows data fitted with a single binding site model for GML and glycerol.

HSA has diverse physiological roles including plasma oncotic pressure regulation and transport of both exogenous and endogenous ligands such as fatty acids [18]. HSA has seven fatty acid binding sites with three high affinity sites characterized by NMR [27]. Therefore HSA potentially binds to GML, a fatty acid monoester, and alters the ability of GML to inhibit human T cell function. Due to the chemical composition of GML, we hypothesize that HSA interacts directly with GML to selectively restore GML mediated T cell defects. Thus, we examined the binding between GML and albumin proteins using isothermal titration calorimetry (ITC). The binding reaction between GML and HSA is exothermic, with a ΔH of ~-3 kcal/mol, and a low micromolar affinity of ~1.4 μM (Table 1). Additionally, GML binds to HSA at a stoichiometry of approximately 3:1, suggesting that GML binds to all three lipid binding sites on HSA (Table 1 and Fig 1A). The lack of an integer number for the experimental stoichiometry is due to error in the measurement of the inactive fraction of each serum albumin and/or concentrations of GML. The affinity and stoichiometry between GML and HSA are consistent with values from other medium chain fatty acids with albumin [28–30]. In comparison, GML binds to albumins from other mammals with similar ΔH and affinity values. GML binds to bovine serum albumin (BSA), pig serum albumin (PSA), rabbit serum albumin (RSA) and mouse serum albumin (MSA) with ΔH values that range between -2.0 to -4.9 kcal/mol and affinities that range between Kd at 2.2 μM and 6.3 μM (Table 1 and S1 Fig). The stoichiometry for the interaction of GML with RSA, PSA and MSA was approximately 3:1, similar to the stoichiometry of the HSA-GML interaction. In contrast, GML bound to BSA with a stoichiometry of 2:1, which corresponds with presence of 2 fatty acid binding sites on BSA [31]. Together, these data show that GML can interact with serum albumins from multiple species with similar affinities, stoichiometries and thermodynamic constants.

**Table 1:** Thermodynamics, stoichiometry, and dissociation constant of albumin with GML, lauric acid or glycerol.

	ΔH (kcal/mol)	n	Kd (μM)
GML + HSA	-3.0 ± 1.1	2.6 ± 0.9	1.4 ± 0.95
GML + BSA	-4.5 ± 0.77	1.7 ± 0.7	2.2 ± 1.1
GML + PSA	-2.0 ± 0.63	3.1 ± 0.2	4.5 ± 1.6
GML + RSA	-4.5 ± 0.61	2.8 ± 0.6	5.4 ± 1.3
GML + MSA	-4.9 ± 1.1	2.6 ± 0.3	6.3 ± 2.7
LA + HSA	-24 ± 0.63	1.1 ± 0.4	4.9 ± 1.1
Glycerol + HSA	N.D.	N.D.	>50

**Table 1:** Thermodynamics, stoichiometry, and dissociation constant of the interaction of GML, lauric acid (LA) or glycerol with human serum albumin (HSA), bovine serum albumin (BSA), pig serum albumin (PSA), rabbit serum albumin (RSA), and mouse serum albumin (MSA). Data generated from ITC experiments were fitted into a single site binding model provided within the ITC software analysis package. Three independent experiments were averaged to produce above values for enthalpy (ΔH), stoichiometry (n), and dissociation constant (Kd). N.D. means not detectable.

Additionally, we investigated whether the 12 carbon acyl chain or the glycerol head group is the principle structural component of GML that binds albumin. To this end, we used ITC to measure the binding affinity between HSA with lauric acid (LA) and glycerol, the 12 carbon saturated acyl chain component of GML and the head group of GML respectively. We found that LA binds to HSA in a similar affinity as GML to HSA with a Kd in the low micromolar range, 4.9 μM. However we found that the stoichiometry of LA to HSA at a 1:1 ratio, which is different from GML to HSA. Moreover, LA binding to HSA is much more exothermic, with an enthalpy value of -24 kcal/mol compared to -3 kcal/mol value observed in the GML and HSA reactions (Table 1 and Fig 1B). In contrast, glycerol did not have measurable binding reaction with HSA (Table 1 and Fig 1C). Thus, GML binds to HSA with a relatively strong affinity via its 12 carbon acyl side chain with glycerol head group being a major determinant of the stoichiometry and thermodynamics of binding.

Using the measured dissociation constant and stoichiometry, we modeled the association between GML and HSA at various physiological concentrations using standard mass action equations. While the presence of other ligands would increase the proportion of free floating GML unbound to albumin, we assumed there were no other albumin ligands present in the system to simplify our calculations. At the GML concentrations used clinically and in this manuscript (10 and 20 μg/ml), >90% of GML molecules are bound to HSA at all HSA concentrations (S2 Fig). It was only when concentrations of GML reached 100–500 μg/ml that substantial portions of GML were not bound by HSA at all concentrations. However, the percentage of HSA bound to GML versus unbound HSA varies drastically with HSA concentration. At the 0.2% HSA concentration, 38% and 75% of HSA is bound to 10 and 20 μg/ml of GML respectively (S2 Fig). In contrast, in a 1% HSA solution only 7.9% and 15.9% of HSA is bound to 10 and 20 μg/ml of GML respectively. Finally at a 2% HSA concentration, <10% of HSA is bound to 10 and 20 μg/ml of GML (S2 Fig). In all, HSA had a strong association with GML with an expected stoichiometry. At the measured affinity and stoichiometry, the percentage of HSA bound to GML is highly dependent on the overall HSA concentration.

### HSA partially rescues downstream phosphorylation of AKT in GML treated cells

**Fig 2:**
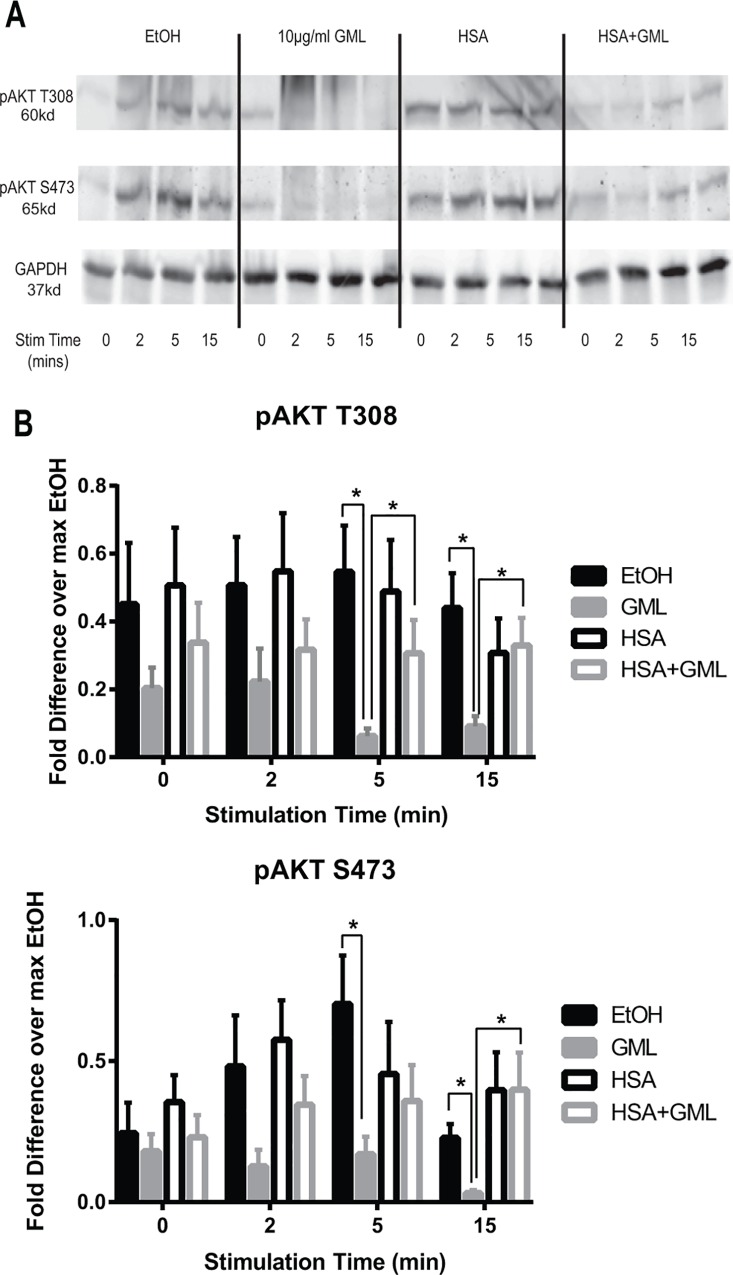
HSA restores AKT phosphorylation at both threonine 308 and serine 473 residues in GML treated cells. APBTs were treated with 0.1% ethanol vehicle control in serum free media (solid black lines), 10 μg/ml GML in serum free media (solid grey lines), 0.1% ethanol vehicle control in 1% HSA (dotted black lines), or 10 μg/ml GML in 1% HSA (dotted grey lines). Cells were stimulated by crosslinking 2 μg/ml of anti-CD3 for various times. Phosphorylation of AKT at threonine 308 and serine 473 were assessed by immunoblotting. Representative blots **(A)** and immunoblot quantification **(B)** are shown from 5 independent experiments with different individual donors. # denotes p<0.05 in Student t’s test comparing ethanol and GML treated cells in serum free media. * denotes p<0.05 in Student t’s test comparing GML treated cells in serum free RPMI with GML treated cells in 1% HSA.

AKT is a key signaling protein in human T cells with many physiological roles including cellular proliferation, survival, metabolism, and T cell development [32]. Activation of AKT kinase activity requires the upstream activation of PI3K and subsequently phosphorylation of AKT at both threonine 308 and serine 473 sites [33]. GML treated cells have attenuated TCR-induced PI3K and AKT phosphorylation [17]. We have shown above that HSA binds to GML with strong affinity. Because of this, HSA could suppress the ability of GML to inhibit human T cell signaling and function. Hence, we asked whether HSA is capable of reversing the suppressed PI3K-AKT signaling axis in GML treated cells. To this end, we treated APBTs with GML or ethanol solvent control in serum free media or media supplemented with 1% HSA. To our surprise, HSA alone decreased the phosphorylation of PI3K p85 regulatory domain following TCR stimulation, suggesting that HSA has independent effects on PI3K phosphorylation (S3 Fig). We also examined the site specific phosphorylation of AKT at both T308 and Y473 residues in the presence of GML with or without HSA. As expected, APBTs treated with GML alone have drastically suppressed AKT T308 and S473 phosphorylation that become statistically significant at 5 and 15 minutes post stimulation compared to ethanol control (Fig 2, compare solid black (EtOH control) with solid gray (GML Treatment) lines). Moreover APBTs in the presence of HSA alone did not have altered AKT phosphorylation at baseline and at all time points post stimulation compared to ethanol control (Fig 2, compare solid (EtOH control) and dotted (HSA alone treatment) black lines). APBTs in the presence of both HSA and GML have a modest increase in the phosphorylation of both residues post stimulation compared to GML treated cells that is below phosphorylation levels in the ethanol control. AKT T308 phosphorylation in HSA and GML treated APBTs become significantly increased compared to APBTs treated with GML alone at 5 and 15 minutes post stimulation, whereas AKT S473 phosphorylation is significantly increased at 15 minutes post stimulation (Fig 2, compare solid (GML alone) and dotted (HSA + GML treatment) gray lines). Hence, HSA impacts the ability of GML to suppress AKT phosphorylation.

### HSA relieves the disruption of LAT, PLC-γ1, and AKT clustering by GML

**Fig 3:**
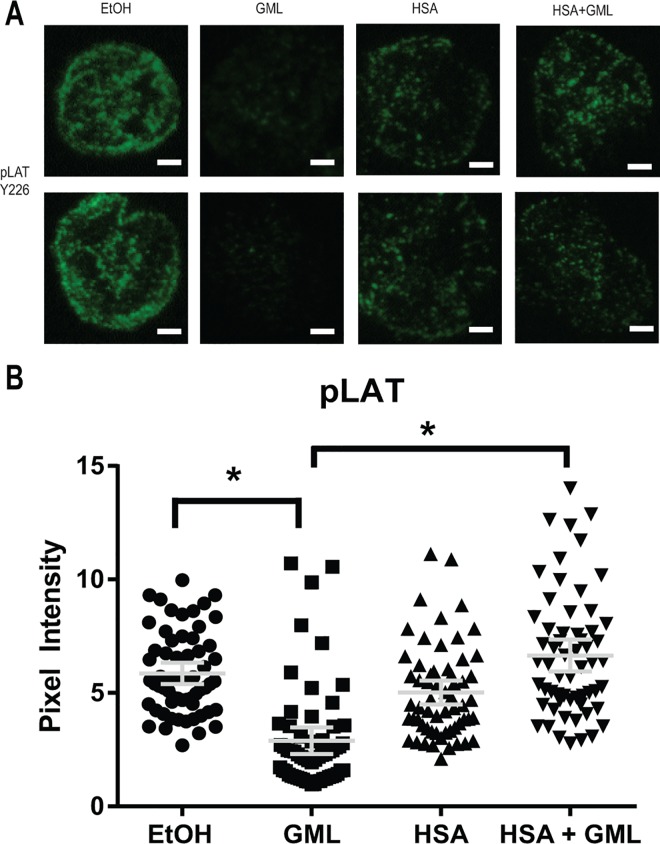
HSA restores GML mediated disruption of LAT microclusters. (**A**). APBTs treated with 0.1% ethanol or 10 μg/ml GML in serum free media as well as 0.1% ethanol or 10 μg/ml GML in 1% HSA were stimulated by plate bound anti-CD3 (2 μg/ml) in glass covered chamber slides. They were then fixed, permeabilized, stained with antibody specific for phosphorylated LAT Y226, and imaged using TIRF microscopy. White bar scale indicates 4 μm in length. (**B**). Pixel intensities of median axis of each cell in images obtained in (**A**) were quantified and averaged using ImageJ. Scatter plot distributions with 95% confidence intervals of 60 cells from 3 independent experiments are shown. * denotes p<0.05 in Student t’s test comparing the identified samples.

**Fig 4:**
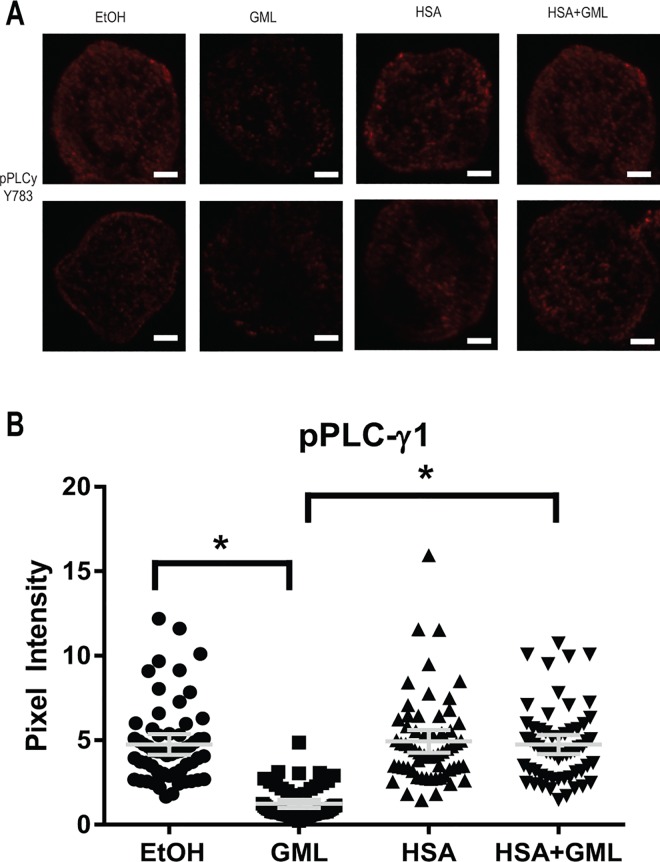
HSA allows for PLC-γ1 microcluster formation in GML treated cells. (**A**). APBTs treated with 0.1% ethanol or 10 μg/ml GML in serum free media as well as 0.1% ethanol or 10μg/ml GML in 1% HSA were stimulated by plate bound anti-CD3 (2 μg/ml) in glass covered chamber slides. They were then fixed, permeabilized, stained with antibody specific for phosphorylated PLC-γ1 Y783, and imaged using TIRF microscopy. White bar scale indicates 4 μm in length. (**B).** Pixel intensities of median axis of each cell in images obtained in (**A**) were quantified and averaged using ImageJ. Scatter plot distributions with 95% confidence intervals of 60 cells from 3 independent experiments are shown. * denotes p<0.05 in Student t’s test comparing the identified samples.

**Fig 5:**
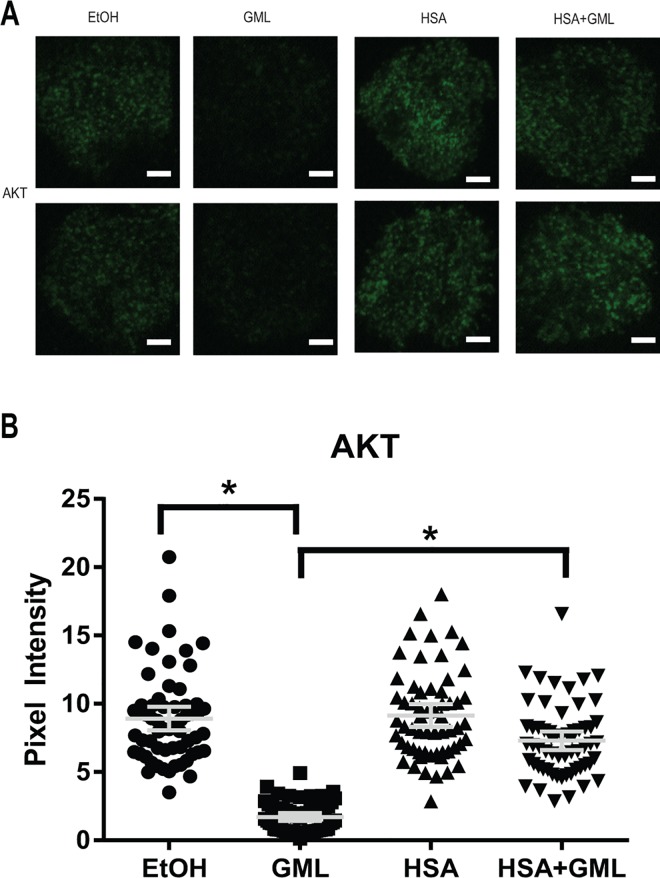
HSA rescues AKT microcluster formation in GML treated cells. (**A**). APBTs treated with 0.1% ethanol or 10 μg/ml GML in serum free media as well as 0.1% ethanol or 10 μg/ml GML in 1% HSA were stimulated by plate bound anti-CD3 (2 μg/ml) in glass covered chamber slides. They were then fixed, permeabilized, stained with antibody specific for total AKT, and imaged using TIRF microscopy. White bar scale indicates 4 μm in length. (**B**). Pixel intensities of median axis of each cell in images obtained in (**A**) were quantified and averaged using ImageJ. Scatter plot distributions with 95% confidence intervals of 60 cells from 3 independent experiments are shown. * denotes p<0.05 in Student t’s test comparing the identified samples.

Activation of the TCR signaling cascade induces the formation of LAT nucleated protein signaling microclusters at the plasma membrane composed of various signaling molecules in close proximity to allow for rapid signal transduction [34–36]. These microclusters are visible under fluorescent microscopy and are highly dependent on TCR activation induced aggregation of smaller nanocluster units of LAT and its signaling partners (<5 LAT molecules) that are not visible under standard fluorescent microscopy [37–39]. We have previously found that GML treated cells have severely reduced LAT, PLC-γ1, and AKT microclusters at the plasma membrane [17]. Because of this, we tested whether HSA alters GML’s impairment of microcluster formation of these molecules. To detect microcluster formation solely in the plasma membrane compartment, we utilized total internal reflection fluorescence (TIRF) microscopy staining for phosphorylated LAT at tyrosine 226. Consistent with our previous report, 10 μg/ml of GML drastically decreased LAT clustering at the membrane. The presence of 1% HSA significantly increased LAT microcluster formation in GML treated cells to levels similar to ethanol and HSA control groups, both visually in representative cells (Fig 3A) and in the average pixel intensity in the cellular median axis of 60 cells (Fig 3B). Similarly, 10 μg/ml of GML alone inhibits PLC-γ1 (Fig 4) and AKT (Fig 5) clustering and the addition of 1% HSA in GML treated cells relieves this inhibition (Fig 4 and Fig 5). Overall, these results show that HSA restores the formation of signaling protein microclusters at the plasma membrane.

### HSA restore cytokine production in GML treated human T cells

**Fig 6:**
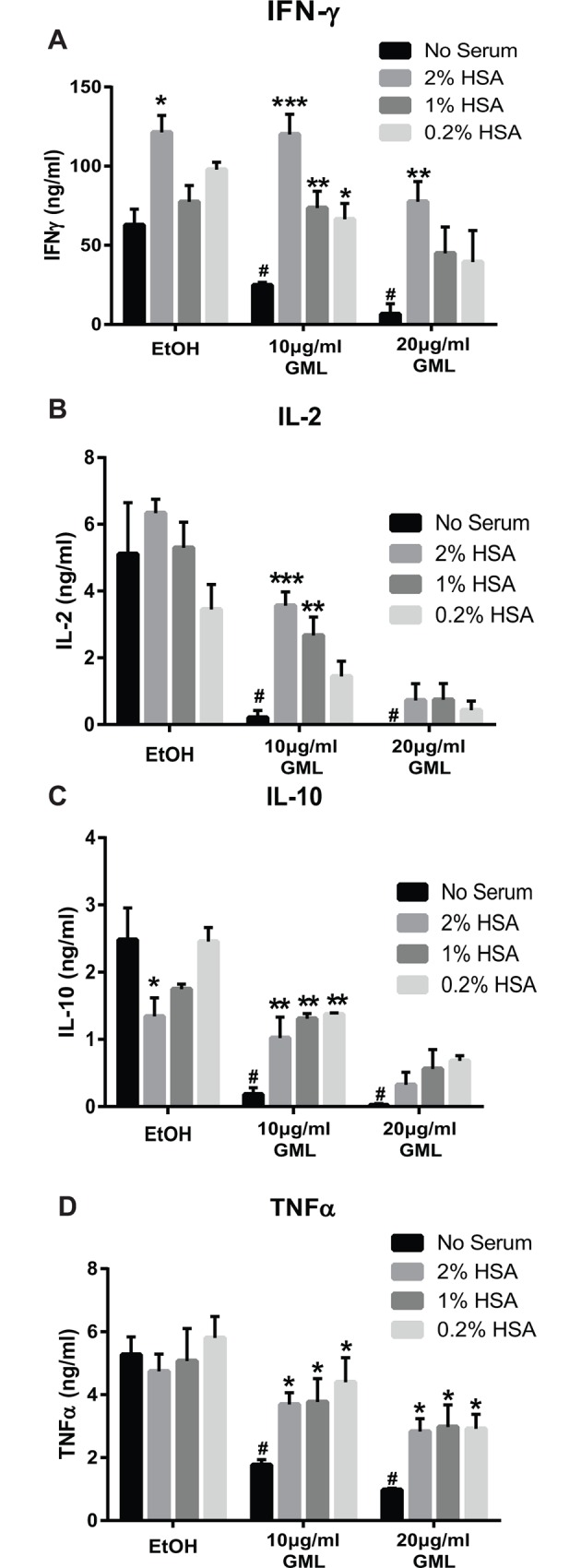
HSA differentially restores cytokine production in GML treated cells. APBTs were suspended in serum free RPMI or RPMI supplemented with 2%, 1% or 0.2% HSA and were treated with 0.2% ethanol vehicle control, 10 μg/ml, or 20 μg/ml of GML. Cells were plated on 2 μg/ml anti-CD3 coated plates for 24 hours. Extracellular cytokine production for **(A)** IFN-γ, **(B)** IL-2, **(C)** IL-10, or **(D)** TNFα was measured by ELISA in 3 independent experiments with different human donors. To statistically confirm that GML is suppressing cytokine production without serum, Student t’s test was done by comparing EtOH treated groups with GML treated groups under the same serum free environment, indicated by the black bars, with # denoting p<0.05. To statistically test for the effects of HSA on GML-induced cytokine production, Student t’s test was done by comparing groups with various concentrations of HSA supplemented media and serum free media with each test group (i.e. the x axis headings EtOH, 10 μg/ml GML, 20 μg/ml GML), with * denoting p<0.05, ** p<0.01, and *** p<0.005.

Our previous data show that HSA binds to GML with strong affinity and alleviates GML-mediated signaling defects in AKT phosphorylation as well as LAT, PLC-γ1, and AKT microcluster formation. Thus, we hypothesized that GML in the presence of HSA is not able to suppress human T cell cytokine production. To test this, we treated APBTs with varying doses of solubilized GML or 0.2% ethanol vehicle control in the presence of 0.1%, 1% and 2% HSA supplemented media and measured TCR-induced cytokine production using ELISA. HSA at these concentrations is within the range of physiological HSA concentrations found in various human tissues [18,19,40,41]. Similar to our previous study, GML in the absence of serum albumin significantly reduced IFNγ, IL-2, IL-10, and TNFα at doses 10 μg/ml or greater compared to APBTs treated with ethanol control in the absence of serum albumin [17] (Fig 6). Curiously, ethanol control with HSA alone induced a significant increase of IFN-γ and decrease of IL-10 compared to ethanol control in the serum free group (Fig 6A and 6C, see the EtOH values). The presence of HSA in addition to GML alleviated GML induced suppression of cytokine production that was distinct for each cytokine tested and was dependent upon the relative concentration of GML and HSA. APBTs in the presence of 2% HSA had significantly restored IFN-γ production at both GML doses compared to GML treatment alone (Fig 6A). However, treatment of APBTs with 1% or 0.2% HSA only had significant restoration of IFN-γ production with 10 μg/ml GML, but not 20 μg/ml of GML (Fig 6A). In contrast, APBTs treated with 10 μg/ml of GML have significantly increased IL-2 production at the HSA concentrations of 2% and 1% but not 0.2% compared to APBTs treated with 10 μg/ml GML in serum free media (Fig 6B). Whereas, APBTs treated with 20 μg/ml of GML did not have significantly increased IL-2 production at all HSA doses compared to APBTs treated in serum free conditions (Fig 6B). Similarly, while APBTs treated with 10 μg/ml of GML in the presence of all doses of HSA have significantly increased IL-10 production compared to control APBTs, treatment with 20 μg/ml GML did not restore IL-10 production when compared to APBTs treated with 20 μg/ml of GML in serum free media across all concentrations of HSA tested (Fig 6C). Finally, APBTs treated with both 10 and 20 μg/ml of GML have significantly increased TNF-α production compared to APBTs treated with 10 and 20 μg/ml of GML in serum free media across all concentrations of HSA (Fig 6D). These observations show that HSA alleviates GML-induced suppression of cytokine production in a distinct manner for each cytokine and is dependent upon the relative ratio of GML and HSA.

## Discussion

GML has potent antimicrobial and immunosuppressive properties; however, no studies have examined how soluble factors at the sites of GML application such as the skin, gut, and vaginal tract modify GML’s activity. Due to the abundance of HSA at these sites and HSA’s known ability to interact with fatty acid compounds, we characterized how HSA alters GML induced T cell suppression. We previously published that ordered lipid domains in the plasma membrane are drastically altered in GML treated cells. Consequently, LAT, PLC-γ1, and AKT nanocluster domains fail to aggregate into microclusters at the plasma membrane. In turn, reduced microcluster formation leads to TCR induced calcium influx, PI3K and AKT activation, and cytokine production [17]. In this study, we found that HSA, PSA, MSA and RSA bind to GML at a stoichiometric ratio of approximately 3:1 at low micromolar affinities, while BSA binds to GML with similar affinities but a stoichiometry of approximately 2:1. The error associated with the stoichiometry values is due to the inability to accurately measure the concentration of the active components of serum albumins and GML. However, the measured stoichiometries correspond with the number of fatty acid binding sites determined by previous studies [27,31]. The 12 carbon acyl chain is the main structural component of GML required for the high affinity binding to HSA, but the glycerol head group of GML plays a role in determining the stoichiometry and thermodynamic constants of this association.

Due to the promiscuous nature of HSA to interact with the dyes used for the calcium influx and lipid order/disorder assays, we were unable to test how HSA affects GML-mediated disruption of membrane lipid dynamics and TCR induced calcium influx. However, we did observe that HSA reversed GML mediated inhibition of membrane clustering of LAT, PLC-γ1, and AKT and partially rescues GML induced suppression of AKT phosphorylation. Finally, HSA restored cytokine production in GML treated T cells, namely IFN-γ, IL-10, TNFα, and IL-2, to various degrees depending on the cytokine. Interestingly, HSA alleviated the suppressed production of inflammatory cytokines, IFN-γ and TNF-α, at a lower ratio of HSA:GML concentrations compared to the immunomodulatory cytokines IL-2 and IL-10. This suggests that HSA is more potent at restoring APBT-induced inflammatory cytokines than immunomodulatory cytokines.

We believe that GML directly binds to HSA in a similar fashion with how HSA binds to its other binding partners. The release of GML induced T cell suppressive effects by albumin is consistent with how albumin interacts with other ligands. HSA binds to warfarin, azapropazone, furosemide, sulfisoxazole, diflunisal, etodolac, and lomefloxacin at affinities similar to GML, with dissociation constants between 2 to 6 μM [42–49]. The degree of association between HSA and its ligands has profound impact on the pharmaceutical activity of those compounds in that increasing concentrations of drug molecules bound to albumin result in decreased drug activity and vice versa. For example, the addition of aspirin affects the binding between HSA and its endogenous ligand bilirubin [50]. Moreover, direct drug displacement of albumin-bound warfarin and sulfonamide antibiotics by other albumin binding compounds, which increases the pool of albumin free warfarin and sulfonamides, decreases the drugs’ effective dosage and potentiates possible toxic side effects [51–53]. Similarly, we believe that the relative concentrations of albumin bound GML vs. albumin free GML dictate how albumin alters the GML induced human T cell suppression. Higher concentrations of albumin remove the vast majority of free GML molecules in solution, resulting in a minimal concentration of free floating, biologically active GML molecules. In contrast, while lower concentrations of albumin is also able to bind to GML molecules, the pool of ligand free albumin is limited, resulting in an accumulation of unbound, biologically active GML molecules as they dissociate from albumin at equilibrium. Moreover, albumin is plausibly bound to a variety of fatty acids and other compounds in a physiological setting. The amount of free floating, active GML molecules would depend heavily on the relative affinity of albumin and GML compared to albumin and other substrates and the amount of HSA present. Thus, we believe that GML is only able to fully suppress human T cells when it is free floating and not bound to albumin. This fits with our data that APBTs treated with 2% albumin and 10 μg/ml of GML, and therefore a high albumin vs. GML ratio, have completely alleviated GML-mediated suppression of IFN-γ, IL-2, IL-10, and TNF-α production. On the other hand, APBTs treated with 0.2% albumin and 20 μg/ml of GML, a much lower albumin vs. GML ratio, resulted in an intermediate phenotype with restored production of the inflammatory cytokine TNFα but not the immunomodulatory cytokines IL-2 and IL-10. Altogether, the concentration of free GML in solution, as determined by the amount of HSA, is critical for the biological activity of GML.

Curiously, HSA appears to have independent effects on T cell responses. Contrary to the classical belief that HSA is an immunologically inert protein, some evidence suggests that HSA alone is able to modulate immune function and signaling. Therapeutic HSA preparations increased the expression of the activation markers MHC-II, CIITA, and H2-M in mice monocytic cells as well as HLA-DR in human monocytic cells, suggesting that HSA may enhance the function of antigen presenting cells [54]. Moreover, HSA stimulates the activation of c-Src kinase and subsequently the MAP kinase and NF-κB pathways to increase VCAM1 expression in endothelial cells, a cellular regulator of the immune response [55]. Additionally, albumin and/or albumin protein fragments may alter human T cell signaling and function. Aspartyl-alanyl diketopiperazine, a cyclized by-product molecule generated by albumin cleavage, suppresses T cell TNF-α and IFN-γ production and MAP kinase cascade activation [56,57]. In this study, we found that human T cells had enhanced IFN-γ and to a lesser extent, reduced IL-10 production compared to the ethanol control cells without HSA. Moreover, we found that HSA alone suppressed TCR-induced phosphorylation of p85 regulatory domain of PI3K (S3 Fig). Albumin may also have additional indirect mechanisms that alter human T cell activation. We observed that T cells in the presence of HSA preparations that were not fatty acid free have altered cytokine production compared to fatty acid free HSA, suggesting that HSA may bind to other fatty acids and ligands that alters human T cells (S3 Fig). Taken together, albumin alone may have additional unexplored effects on human T cell signaling and function. Despite this, our data clearly shows that HSA alleviates the suppression of human T cells by GML.

Collectively, we found that HSA is a powerful modulator of human T cell suppression by GML which provide important contextual insight on how GML exert its immunosuppressive properties. When administered systemically into the circulatory system, where plasma albumin concentrations range between 35 to 50 g/L, GML will encounter abundant HSA at concentrations higher than the measured HSA-GML Kd value [18,58], especially in light of the fact that GML has an aqueous solubility cap of 100 μg/ml under 37°C. Utilizing the observed Kd value of 1.4 μM to calculate the concentration of the GML-HSA complex and comparing it to free GML molecules, we found that >99% of GML molecules is bound to albumin at concentrations where GML is soluble (S2 Fig). Based on our findings, GML is not expected to cause systemic T cell suppression as it would bind to HSA and be sequestered away from T cells entirely. Moreover, any free floating or albumin dissociated GML molecules would rapidly encounter albumin not bound to GML due to the large amount of albumin molecules present in the total volume of serum.

Similarly, albumin is highly concentrated in the lymphatic fluid, where its concentration ranges from 40% to 90% of the serum concentration, and dynamic lymphatic flow allows for a large pool of albumin molecules to encounter GML [40]. Under similar conditions where the HSA concentrations are higher than the HSA-GML Kd value, we again found that >99% of albumin is bound to GML in this scenario (S2 Fig). From our observations, systemic GML is not expected to hinder T cell activation and follicular T cell functions in the lymph node. Additionally, topical GML in gel formulations with solubility up to 50 mg/ml can readily come into contact with highly concentrated albumin in superficial skin wounds, where exudate fluids contain albumin at concentrations ranging from 10 to 30g/L, which is in excess of the HSA-GML Kd value [3,19]. This pool of albumin is replenished from the plasma via capillary leakage, albeit at a smaller total plasma volume than systemic circulation [19]. We calculate that >90% of GML at doses less than 500 μg/ml will be bound to HSA. GML at doses between 500 to 5000 μg/ml will result in an equilibrium state where the concentration of GML-HSA complexes is within one order of magnitude of free GML molecules. In contrast, <2% of GML molecules will be bound to HSA at doses of GML greater than 5000 μg/ml (S2 Fig). Thus, GML at therapeutic doses between 500 to 5000 μg/ml is not expected to be completely inhibit pathogen clearance or immune function, but instead may result in an intermediate phenotype characterized by a cytokine milieu with intact inflammatory but suppressed immunomodulatory cytokines.

In contrast, low levels of albumin without a functional reserve are present in the urine, vaginal secretions, cerebrospinal fluid, tears, bile, sweat, gastric juice, eye, and other tissues. In these tissues, HSA concentrations are within an order of magnitude to the measured HSA-GML Kd value at the higher range of physiological values and drops below the Kd in the lower range [41]. We calculate that at the relevant HSA concentration in these sites, GML at doses less than 20 μg/ml would be mostly bound to albumin and albumin-free GML would increase rapidly at doses greater than 20 μg/ml (S2 Fig). Therefore, albumin at these sites is not expected to completely prevent T cell suppression by GML at therapeutic doses greater than 20 μg/ml, allowing GML to suppress inflammation locally in these tissues. This is especially important in the context of topical GML application in the vaginal tract to remediate menstrual associated toxic shock syndrome, where GML would inhibit both gram positive bacteria virulence and unchecked superantigen induced T cell activation. Overall, we demonstrate that HSA directly binds and sequesters GML molecules away from T cells and the concentration of free floating GML molecules is a major determinant of GML’s ability to suppress human T cell signaling and function. These observations provide insight on how host factors modify GML’s immunosuppressive properties and the context for how GML achieves T cell suppression.

## Supporting Information

S1 Fig ITC curves of GML with various mammalian albumins. 150μM of GML was injected incrementally into the sample chamber containing 5μM of mammalian albumins. Top graph shows the change of power with each GML injection and bottom graph shows data fitted with a single binding site model for **(A)** bovine serum albumin (BSA), **(B)** pig serum albumin (PSA), **(C)** rabbit serum albumin (RSA), and **(D)** mouse serum albumin (MSA).
(PDF)

S2 Fig Binding curves of GML with HSA. The percentage of GML molecules bound to HSA **(A)** and conversely HSA bound to GML **(B)** were calculated using the Kd value between GML and HSA in. Curves for 4%, 2%, 1%, 0.2%, and 0.0002% HSA are shown with open diamond, circle, square, triangle, and closed diamond respectively.
(PDF)

S3 Fig HSA and non-fatty acid free HSA have independent effects on T cell signaling and function. **(A)** APBTs were treated with 0.1% ethanol vehicle control in serum free media, 10 μg/ml GML in serum free media, 0.1% ethanol vehicle control in 1% fatty acid free-HSA, or 10 μg/ml GML in 1% fatty acid free-HSA. Cells were stimulated by crosslinking 2 μg/ml of anti-CD3 for various times. Phosphorylation of p85 regulatory domain of PI3K was assessed by immunoblotting with representative blot shown. **(B)** APBTs were suspended in serum free RPMI or RPMI supplemented with 3% non-fatty acid free HSA and were treated with 0.2% ethanol vehicle control, 10 μg/ml, or 20 μg/ml of GML. Cells were plated on 2 μg/ml anti-CD3 coated plates for 24 hours. Extracellular cytokine production for IFN-γ (top left), IL-2 (top right), IL-10 (bottom left), or TNFα (bottom right) was measured by ELISA.
(PDF)

## References

[1] Schlievert PM, Deringer JR, Kim MH, Projan SJ, Novick RP (1992) Effect of glycerol monolaurate on bacterial growth and toxin production. Antimicrob Agents Chemother 36: 626–631. 1622174 10.1128/aac.36.3.626 PMC190568

[2] Projan SJ, Brown-Skrobot S, Schlievert PM, Vandenesch F, Novick RP (1994) Glycerol monolaurate inhibits the production of beta-lactamase, toxic shock toxin-1, and other staphylococcal exoproteins by interfering with signal transduction. J Bacteriol 176: 4204–4209. 8021206 10.1128/jb.176.14.4204-4209.1994 PMC205630

[3] Schlievert PM, Peterson ML (2012) Glycerol monolaurate antibacterial activity in broth and biofilm cultures. PLoS One 7: e40350 10.1371/journal.pone.0040350 22808139 PMC3394780

[4] Strandberg KL, Peterson ML, Lin YC, Pack MC, Chase DJ, Schlievert PM (2010) Glycerol monolaurate inhibits Candida and Gardnerella vaginalis in vitro and in vivo but not Lactobacillus. Antimicrob Agents Chemother 54: 597–601. 10.1128/AAC.01151-09 20008774 PMC2812150

[5] Clarke NM, May JT (2000) Effect of antimicrobial factors in human milk on rhinoviruses and milk-borne cytomegalovirus in vitro. J Med Microbiol 49: 719–723. 10.1099/0022-1317-49-8-719 10933257

[6] Li Q, Estes JD, Schlievert PM, Duan L, Brosnahan AJ, Southern PJ, et al (2009) Glycerol monolaurate prevents mucosal SIV transmission. Nature 458: 1034–1038. 10.1038/nature07831 19262509 PMC2785041

[7] 21.CFR.184.1505 (2010) Title 21. Food and drugs. Chapter I. Food and Drug Administration. Subchapter B. Food for human consumption. Part 184. Direct food substances affirmed as generally recognized as safe. Subpart B. Listing of specific substances affirmed as GRAS. 21 CFR 184.1505—mono- and diglycerides Code of Federal Regulations.

[8] Hoppe UE, Ulrich; Sauermann, Gerhard; Engel, Walter; Pape, Wolfgang (20. Apr. 1999) Deodorizing and antimicrobial composition for use in cosmetic or topical formulations. United States Beiersdorf Aktiengesellschaft.

[9] Blaszyk M, Holley RA (1998) Interaction of monolaurin, eugenol and sodium citrate on growth of common meat spoilage and pathogenic organisms. Int J Food Microbiol 39: 175–183. 9553796 10.1016/s0168-1605(97)00134-7

[10] Strandberg KL, Peterson ML, Schaefers MM, Case LC, Pack MC, Chase DJ, et al (2009) Reduction in Staphylococcus aureus growth and exotoxin production and in vaginal interleukin 8 levels due to glycerol monolaurate in tampons. Clin Infect Dis 49: 1711–1717. 10.1086/644614 19863450

[11] Haase AT, Rakasz E, Schultz-Darken N, Nephew K, Weisgrau KL, Reilly CS, et al (2015) Glycerol Monolaurate Microbicide Protection against Repeat High-Dose SIV Vaginal Challenge. PLoS One 10: e0129465 10.1371/journal.pone.0129465 26057743 PMC4461171

[12] Mueller EA, Schlievert PM (2015) Non-aqueous glycerol monolaurate gel exhibits antibacterial and anti-biofilm activity against Gram-positive and Gram-negative pathogens. PLoS One 10: e0120280 10.1371/journal.pone.0120280 25799455 PMC4370562

[13] Preuss HG, Echard B, Dadgar A, Talpur N, Manohar V, Enig M, et al (2005) Effects of Essential Oils and Monolaurin on Staphylococcus aureus: In Vitro and In Vivo Studies. Toxicol Mech Methods 15: 279–285. 10.1080/15376520590968833 20021093

[14] Manohar V, Echard B, Perricone N, Ingram C, Enig M, Bagchi D, et al (2013) In vitro and in vivo effects of two coconut oils in comparison to monolaurin on Staphylococcus aureus: rodent studies. J Med Food 16: 499–503. 10.1089/jmf.2012.0066 23767861

[15] Witcher KJ, Novick RP, Schlievert PM (1996) Modulation of immune cell proliferation by glycerol monolaurate. Clin Diagn Lab Immunol 3: 10–13. 8770497 10.1128/cdli.3.1.10-13.1996 PMC170240

[16] Peterson ML, Schlievert PM (2006) Glycerol monolaurate inhibits the effects of Gram-positive select agents on eukaryotic cells. Biochemistry 45: 2387–2397. 10.1021/bi051992u 16475828 PMC2553893

[17] Zhang MS, Sandouk A, Houtman JC (2016) Glycerol Monolaurate (GML) inhibits human T cell signaling and function by disrupting lipid dynamics. Sci Rep 6: 30225 10.1038/srep30225 27456316 PMC4960522

[18] Peters T (1995) All about Albumin: Biochemistry, Genetics, and Medical Applications London, UK: Academic Press.

[19] James TJ, Hughes MA, Cherry GW, Taylor RP (2000) Simple biochemical markers to assess chronic wounds. Wound Repair Regen 8: 264–269. 11013017 10.1046/j.1524-475x.2000.00264.x

[20] Evans TW (2002) Review article: albumin as a drug—biological effects of albumin unrelated to oncotic pressure. Aliment Pharmacol Ther 16 Suppl 5: 6–11. 12423448 10.1046/j.1365-2036.16.s5.2.x

[21] Kragh-Hansen U (1981) Molecular aspects of ligand binding to serum albumin. Pharmacol Rev 33: 17–53. 7027277

[22] Palareti G, Legnani C (1996) Warfarin withdrawal. Pharmacokinetic-pharmacodynamic considerations. Clin Pharmacokinet 30: 300–313. 8983860 10.2165/00003088-199630040-00003

[23] Bilal MY, Houtman JC (2015) GRB2 Nucleates T Cell Receptor-Mediated LAT Clusters That Control PLC-gamma1 Activation and Cytokine Production. Front Immunol 6: 141 10.3389/fimmu.2015.00141 25870599 PMC4378308

[24] Chapman NM, Yoder AN, Barbon KM, Bilal MY, Connolly SF, Houtman JC (2015) Proline-rich tyrosine kinase 2 controls PI3-kinase activation downstream of the T cell antigen receptor in human T cells. J Leukoc Biol 97: 285–296. 10.1189/jlb.2A1013-568RRR 25387834 PMC4304419

[25] Cruz-Orcutt N, Vacaflores A, Connolly SF, Bunnell SC, Houtman JC (2014) Activated PLC-gamma1 is catalytically induced at LAT but activated PLC-gamma1 is localized at both LAT- and TCR-containing complexes. Cell Signal 26: 797–805. 10.1016/j.cellsig.2013.12.022 24412752 PMC3935424

[26] Moosavi-Movahedi AA, Chamani J, Gharanfoli M, Hakimelahi GH (2004) Differential scanning calorimetric study of the molten globule state of cytochrome c induced by sodium n-dodecyl sulfate. Thermochimica Acta 409: 137–144.

[27] Hamilton JA (2013) NMR reveals molecular interactions and dynamics of fatty acid binding to albumin. Biochim Biophys Acta 1830: 5418–5426. 10.1016/j.bbagen.2013.08.002 23939311

[28] Hamilton JA, Cistola DP, Morrisett JD, Sparrow JT, Small DM (1984) Interactions of myristic acid with bovine serum albumin: a 13C NMR study. Proc Natl Acad Sci U S A 81: 3718–3722. 6587386 10.1073/pnas.81.12.3718 PMC345290

[29] Simard JR, Zunszain PA, Ha CE, Yang JS, Bhagavan NV, Petitpas I, et al (2005) Locating high-affinity fatty acid-binding sites on albumin by x-ray crystallography and NMR spectroscopy. Proc Natl Acad Sci U S A 102: 17958–17963. 10.1073/pnas.0506440102 16330771 PMC1312385

[30] Reed RG, Feldhoff RC, Clute OL, Peters T Jr. (1975) Fragments of bovine serum albumin produced by limited proteolysis. Conformation and ligand binding. Biochemistry 14: 4578–4583. 1237311 10.1021/bi00692a004

[31] Reynolds J, Herbert S, Steinhardt J (1968) The binding of some long-chain fatty acid anions and alcohols by bovine serum albumin. Biochemistry 7: 1357–1361. 5677825 10.1021/bi00844a016

[32] Fayard E, Xue G, Parcellier A, Bozulic L, Hemmings BA (2010) Protein kinase B (PKB/Akt), a key mediator of the PI3K signaling pathway. Curr Top Microbiol Immunol 346: 31–56. 10.1007/82_2010_58 20517722

[33] Alessi DR, Cohen P (1998) Mechanism of activation and function of protein kinase B. Curr Opin Genet Dev 8: 55–62. 9529606 10.1016/s0959-437x(98)80062-2

[34] Bunnell SC, Hong DI, Kardon JR, Yamazaki T, McGlade CJ, Barr VA, et al (2002) T cell receptor ligation induces the formation of dynamically regulated signaling assemblies. J Cell Biol 158: 1263–1275. 10.1083/jcb.200203043 12356870 PMC2173229

[35] Yokosuka T, Sakata-Sogawa K, Kobayashi W, Hiroshima M, Hashimoto-Tane A, Tokunaga M, et al (2005) Newly generated T cell receptor microclusters initiate and sustain T cell activation by recruitment of Zap70 and SLP-76. Nat Immunol 6: 1253–1262. 10.1038/ni1272 16273097

[36] Balagopalan L, Kortum RL, Coussens NP, Barr VA, Samelson LE (2015) The Linker for Activation of T cells (LAT) signaling hub: from signaling complexes to microclusters. J Biol Chem. 10.1074/jbc.R115.665869 PMC4646300 26354432

[37] Balagopalan L, Barr VA, Kortum RL, Park AK, Samelson LE (2013) Cutting edge: cell surface linker for activation of T cells is recruited to microclusters and is active in signaling. J Immunol 190: 3849–3853. 10.4049/jimmunol.1202760 23487428 PMC4144449

[38] Coussens NP, Hayashi R, Brown PH, Balagopalan L, Balbo A, Akpan I, et al (2013) Multipoint binding of the SLP-76 SH2 domain to ADAP is critical for oligomerization of SLP-76 signaling complexes in stimulated T cells. Mol Cell Biol 33: 4140–4151. 10.1128/MCB.00410-13 23979596 PMC3811887

[39] Sherman E, Barr V, Manley S, Patterson G, Balagopalan L, Akpan I, et al (2011) Functional nanoscale organization of signaling molecules downstream of the T cell antigen receptor. Immunity 35: 705–720. 10.1016/j.immuni.2011.10.004 22055681 PMC3225724

[40] Berson SA, Yalow RS, Schreiber SS, Post J (1953) Tracer experiments with I131 labeled human serum albumin: distribution and degradation studies. J Clin Invest 32: 746–768. 10.1172/JCI102789 13069623 PMC438398

[41] Bruschi M, Santucci L, Candiano G, Ghiggeri GM (2013) Albumin heterogeneity in low-abundance fluids. The case of urine and cerebro-spinal fluid. Biochim Biophys Acta 1830: 5503–5508. 10.1016/j.bbagen.2013.04.026 23628703

[42] Yamasaki K, Chuang VT, Maruyama T, Otagiri M (2013) Albumin-drug interaction and its clinical implication. Biochim Biophys Acta 1830: 5435–5443. 10.1016/j.bbagen.2013.05.005 23665585

[43] Yamasaki K, Maruyama T, Kragh-Hansen U, Otagiri M (1996) Characterization of site I on human serum albumin: concept about the structure of a drug binding site. Biochim Biophys Acta 1295: 147–157. 8695640 10.1016/0167-4838(96)00013-1

[44] Kragh-Hansen U (1988) Evidence for a large and flexible region of human serum albumin possessing high affinity binding sites for salicylate, warfarin, and other ligands. Mol Pharmacol 34: 160–171. 3412320

[45] Takamura N, Maruyama T, Chosa E, Kawai K, Tsutsumi Y, Uryu Y, et al (2005) Bucolome, a potent binding inhibitor for furosemide, alters the pharmacokinetics and diuretic effect of furosemide: potential for use of bucolome to restore diuretic response in nephrotic syndrome. Drug Metab Dispos 33: 596–602. 10.1124/dmd.104.002782 15640375

[46] Anton AH (1973) Drug-protein binding. IV. Modulation of binding. Increasing activity of sulfonamides with displacing agents: a review. Ann N Y Acad Sci 226: 273–292. 4588442 10.1111/j.1749-6632.1973.tb20490.x

[47] Verbeeck RK, Boel A, Buntinx A, De Schepper PJ (1980) Plasma protein binding and interaction studies with diflunisal, a new salicylate analgesic. Biochem Pharmacol 29: 571–576. 7370051 10.1016/0006-2952(80)90378-0

[48] Mignot I, Presle N, Lapicque F, Monot C, Dropsy R, Netter P (1996) Albumin binding sites for etodolac enantiomers. Chirality 8: 271–280. 10.1002/(SICI)1520-636X(1996)8:3&lt;271::AID-CHIR7&gt;3.0.CO;2-K 8777148

[49] Vahedian-Movahed H, Saberi MR, Chamani J (2011) Comparison of binding interactions of lomefloxacin to serum albumin and serum transferrin by resonance light scattering and fluorescence quenching methods. J Biomol Struct Dyn 28: 483–502. 10.1080/07391102.2011.10508590 21142219

[50] Hosainzadeh A, Gharanfoli M, Saberi M, Chamani J (2012) Probing the interaction of human serum albumin with bilirubin in the presence of aspirin by multi-spectroscopic, molecular modeling and zeta potential techniques: insight on binary and ternary systems. J Biomol Struct Dyn 29: 1013–1050. 10.1080/073911012010525029 22292958

[51] Brodie BB (1965) Displacement of one drug by another from carrier or receptor sites. Proc R Soc Med 58: 946–955. 5854428 10.1177/003591576505811P202 PMC1898665

[52] Tillement JP, Zini R, Mattei C, Singlas E (1973) Effect of phenylbutazone on the binding of vitamin K antagonists to albumin. Eur J Clin Pharmacol 6: 15–18. 4764367 10.1007/BF00561795

[53] Anton AH (1960) The relation between the binding of sulfonamides to albumin and their antibacterial efficacy. J Pharmacol Exp Ther 129: 282–290. 13793859

[54] Aubin E, Roberge C, Lemieux R, Bazin R (2011) Immunomodulatory effects of therapeutic preparations of human albumin. Vox Sang 101: 131–137. 10.1111/j.1423-0410.2011.01475.x 21426357

[55] Baek JE, Yang WS, Chang JW, Kim SB, Park SK, Park JS, et al (2010) Fatty acid-bearing albumin induces VCAM-1 expression through c-Src kinase-AP-1/NF-kB pathways: effect of L-carnitine. Kidney Blood Press Res 33: 72–84. 10.1159/000289576 20197690

[56] Shimonkevitz R, Thomas G, Slone DS, Craun M, Mains C, Bar-Or D (2008) A diketopiperazine fragment of human serum albumin modulates T-lymphocyte cytokine production through rap1. J Trauma 64: 35–41. 10.1097/TA.0b013e3181589ff9 18188096

[57] Bar-Or D, Thomas GW, Bar-Or R, Rael LT, Scarborough K, Rao N, et al (2006) Commercial human albumin preparations for clinical use are immunosuppressive in vitro. Crit Care Med 34: 1707–1712. 10.1097/01.CCM.0000217923.53680.4C 16625113

[58] Lin YC, Schlievert PM, Anderson MJ, Fair CL, Schaefers MM, Muthyala R, et al (2009) Glycerol monolaurate and dodecylglycerol effects on Staphylococcus aureus and toxic shock syndrome toxin-1 in vitro and in vivo. PLoS One 4: e7499 10.1371/journal.pone.0007499 19838303 PMC2759527

