# Supplementary figures and images for: Human Serum Albumin (HSA) Suppresses the Effects of Glycerol Monolaurate (GML) on Human T Cell Activation and Function
Source: PLoS One. 2016 Oct 20;11(10):e0165083. doi: 10.1371/journal.pone.0165083 (PMC5072635; doi:10.1371/journal.pone.0165083)

# Supplementary Figure 1

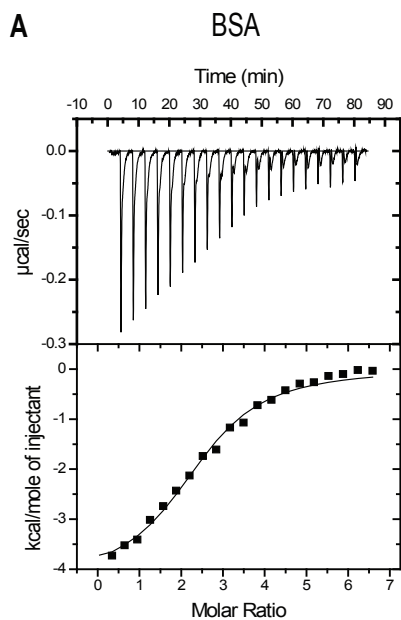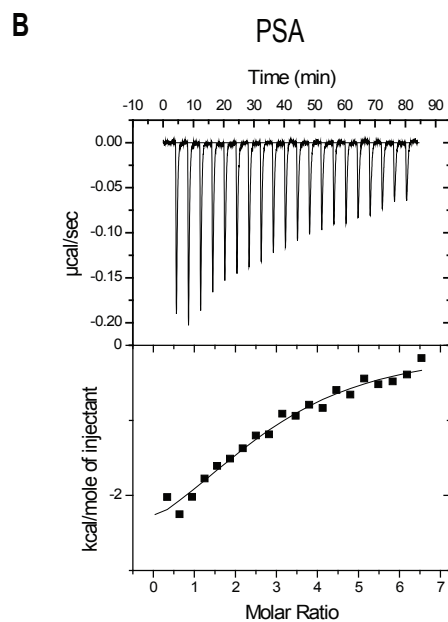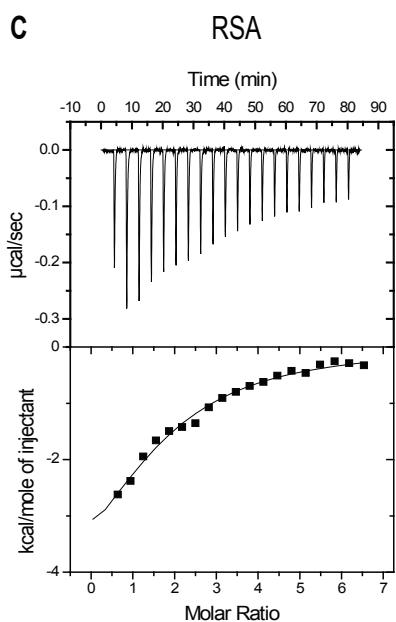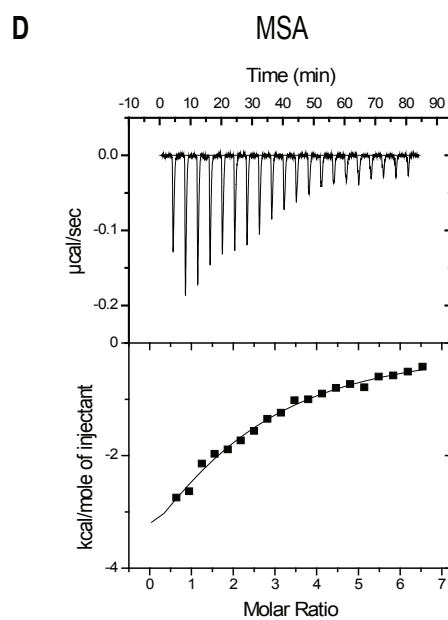

Supplement: S1 Fig — 150μM of GML was injected incrementally into the sample chamber containing 5μM of mammalian albumins. Top graph shows the change of power with each GML injection and bottom graph shows data fitted with a single binding site model for (A) bovine serum albumin (BSA), (B) pig serum albumin (PSA), (C) rabbit serum albumin (RSA), and (D) mouse serum albumin (MSA). (PDF) [file pone.0165083.s001.pdf]

Supplementary Figure 2

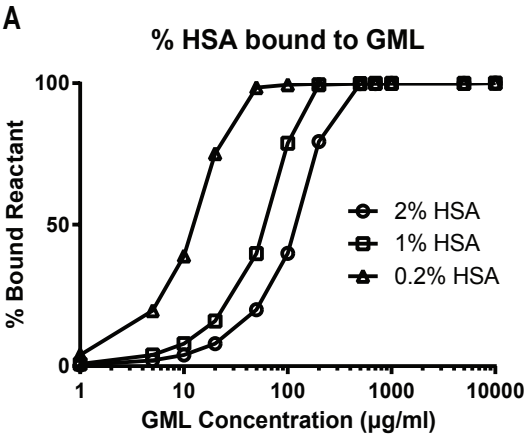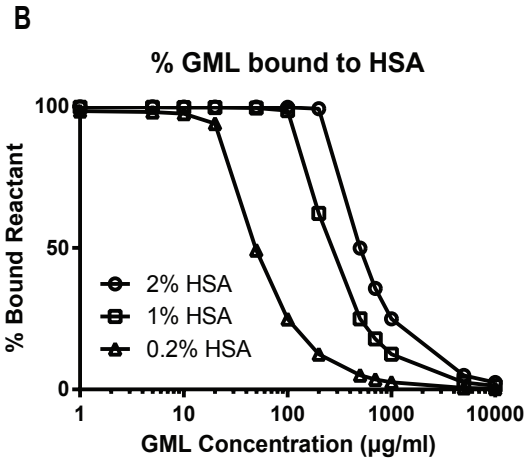

Supplement: S2 Fig — The percentage of GML molecules bound to HSA (A) and conversely HSA bound to GML (B) were calculated using the Kd value between GML and HSA in. Curves for 4%, 2%, 1%, 0.2%, and 0.0002% HSA are shown with open diamond, circle, square, triangle, and closed diamond respectively. (PDF) [file pone.0165083.s002.pdf]

Supplementary Figure 3

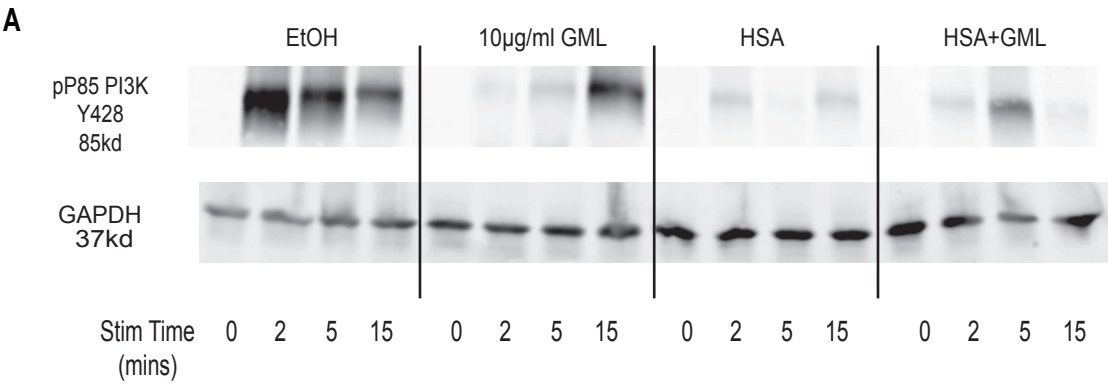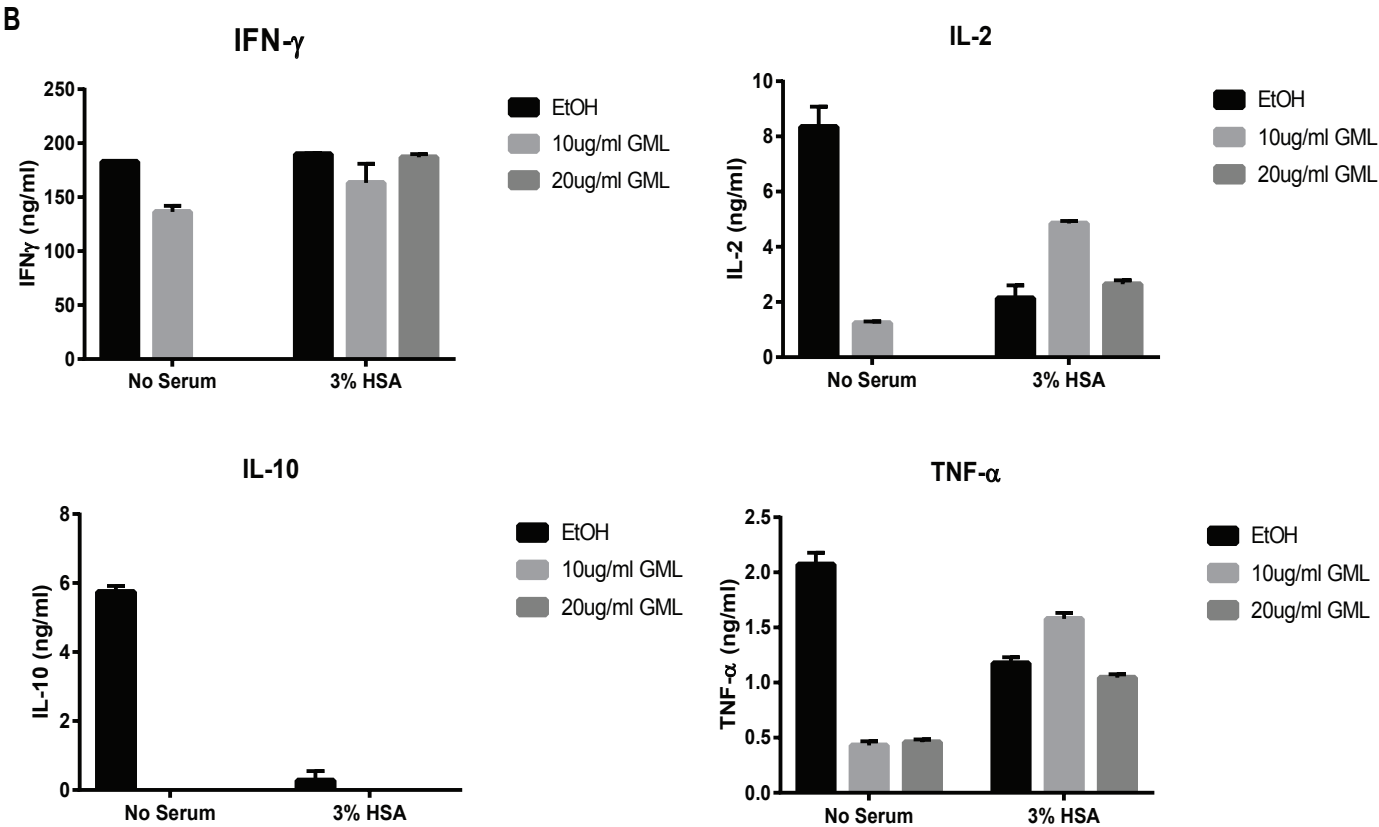

Supplement: S3 Fig — (A) APBTs were treated with 0.1% ethanol vehicle control in serum free media, 10 μg/ml GML in serum free media, 0.1% ethanol vehicle control in 1% fatty acid free-HSA, or 10 μg/ml GML in 1% fatty acid free-HSA. Cells were stimulated by crosslinking 2 μg/ml of anti-CD3 for various times. Phosphorylation of p85 regulatory domain of PI3K was assessed by immunoblotting with representative blot shown. (B) APBTs were suspended in serum free RPMI or RPMI supplemented with 3% non-fatty acid free HSA and were treated with 0.2% ethanol vehicle control, 10 μg/ml, or 20 μg/ml of GML. Cells were plated on 2 μg/ml anti-CD3 coated plates for 24 hours. Extracellular cytokine production for IFN-γ (top left), IL-2 (top right), IL-10 (bottom left), or TNFα (bottom right) was measured by ELISA. (PDF) [file pone.0165083.s003.pdf]
